# Pack-Boriding of Fe-20Cr-5Al Alloy: Nanostructured Boride Layer Formation, Mechanical Performance, and Paradoxical Passivation Loss via Micro-Galvanic Interactions

**DOI:** 10.3390/nano16140870

**Published:** 2026-07-15

**Authors:** Cengiz Temiz, Uğur Öztürk, Seyit Çağlar, Fikret Yılmaz

**Affiliations:** 1 Department of Electronics and Automation, Alaplı Vocational School, Zonguldak Bülent Ecevit University, Zonguldak 67850, Türkiye; 2 Department of Hybrid and Electric Vehicles Technology, Tokat Gaziosmanpaşa University, Tokat 60300, Türkiye; ugur.ozturk@gop.edu.tr; 3 Department of Metallurgical and Materials Engineering, Zonguldak Bülent Ecevit University, Zonguldak 67100, Türkiye; 4 Department of Physics, Tokat Gaziosmanpaşa University, Tokat 60300, Türkiye; fikret.yilmaz@gop.edu.tr

**Keywords:** Fe-20Cr-5Al, pack-boriding, nanocrystalline boride layer, Williamson-Hall analysis, Al-repulsion, corrosion, wear resistance

## Abstract

This study investigates the microstructural evolution, mechanical performance, and electrochemical corrosion behavior of nanocrystalline boride layers formed on an Fe-20Cr-5Al ferritic alloy by pack boriding at 950 °C for 4 h. X-ray diffraction (XRD) and scanning electron microscopy/energy-dispersive X-ray spectroscopy (SEM/EDS) analyses confirmed the formation of a hierarchical boride layer approximately 80–85 μm in thickness, consisting of orthorhombic (Fe,Cr)B and tetragonal (Fe,Cr)_2_B phases at the surface and (Fe,Cr)_23_(C,B)_6_ carboboride phases in the diffusion zone, the latter attributed to the carbon push-ahead mechanism. Rietveld refinement yielded a quantitative phase fraction of 51.9 wt.%. (Fe,Cr)B, 46.1 wt.% Fe_2_B, and 2.0 wt.% (Fe,Cr)_23_(C,B)_6_, with a theoretical boride layer density of 7.40 g cm^−3^. Williamson–Hall analysis yielded an average crystallite size of 50.7 nm and a microstrain of 1.686 × 10^−3^, confirming the nanocrystalline character of the boride phases. Mechanical evaluation revealed a ~9-fold increase in surface hardness in Fe20Cr5Al-B relative to Fe20Cr5Al, reaching 1854 HV (18.18 GPa). Tribological testing demonstrated an ~18-fold reduction in wear rate (from 3.29 × 10^−4^ to 1.82 × 10^−5^ mm^3^/m) and a 14.5% reduction in the coefficient of friction (0.76→0.65), confirming the effectiveness of the boride layer as a tribological barrier. However, electrochemical analyses in 5 wt.% H_2_SO_4_ revealed a paradoxical deterioration in corrosion resistance: despite a noble shift in *E_corr_* from −0.459 to −0.295 V, the corrosion rate increased ~4-fold (from 9.67 × 10^−3^ to 3.83 × 10^−2^ mm/year), driven by Al-repulsion-induced passive film loss and micro-galvanic cell formation through micro-crack and porosity networks. These findings emphasize that while pack-boriding is highly effective for tribological enhancement of FeCrAl alloys, minimizing boride layer defects is essential to achieve concurrent corrosion protection in acidic environments.

## 1. Introduction

Today, nanomaterial production should focus not only on achieving high mechanical performance but also on minimizing environmental impacts, optimizing production processes for energy efficiency, and reducing dependence on critical raw materials [1,2,3,4,5]. Iron-chromium-aluminum (FeCrAl) alloys are ferritic stainless steels with a body-centered cubic (BCC) crystal structure, typically containing 15–25 wt. % chromium and 4–6 wt.% aluminum. These alloys are well recognized for their resistance to high-temperature oxidation and neutron irradiation, and their low thermal expansion coefficient, combined with high electrical resistivity, makes them strategically important across a range of industrial sectors [6,7]. When exposed to elevated temperatures, a thermodynamically stable, dense, and slow-growing α-Al_2_O_3_ (alumina) scale forms on the surface, which is why these alloys are also referred to as alumina-forming ferritic (AFF) steels [8,9]. This alumina scale effectively suppresses oxygen diffusion into the substrate at high temperatures, providing superior oxidation protection compared to chromia-forming counterparts. Owing to these properties, FeCrAl alloys find application in industrial furnace heating elements, catalytic converter substrates in exhaust systems, accident-tolerant fuel (ATF) cladding in nuclear energy, superheater components in chemical looping combustion (CLC) systems, and light water reactors (LWRs) [10].

Despite their excellent oxidation resistance, FeCrAl alloys face notable structural and mechanical limitations under demanding service conditions. In high-temperature steam environments or aggressive acidic media, the protective oxide scale may lose stability, leading to a phenomenon known as breakaway oxidation, an abrupt acceleration of corrosion [11,12]. In nuclear reactor environments, hydrogen generated by water radiolysis can permeate into the alloy matrix, increasing its susceptibility to embrittlement and potentially causing brittle fracture under service loads [13,14,15,16].

Surface degradation from wear and corrosion remains one of the most critical factors limiting the service life of engineering components. To extend component longevity and improve resistance under extreme operating conditions, various surface modification techniques have been developed, including physical vapor deposition (PVD) coatings, nitriding, carburizing, and boriding. These methods aim to enhance surface properties, particularly wear and corrosion resistance, without altering the material’s bulk microstructure [17]. The importance of microstructural optimization in achieving superior tribological performance has also been demonstrated in recent advances in refractory hard materials, including WC-based cermets [18].

Corrosion, one of the leading causes of failure in engineering components, involves the electrochemical degradation of a material through interaction with its environment [19]. Boride layers have been reported to act as physical barriers that isolate the substrate from the surrounding medium, substantially reducing corrosion current density [20]. Although the effect of boriding on corrosion resistance varies with layer morphology and the distribution of alloying elements, boride layers formed under optimized conditions have consistently been reported to provide protective behavior in acidic and corrosive environments [21,22]. Studies on various steel grades have demonstrated that boriding markedly reduces corrosion rates in acidic and chloride-containing media [23,24,25], with borided low-carbon steel surfaces exhibiting significantly superior performance compared with the untreated substrate in acidic solutions [26]. The low thermal conductivity of boride layers also enables their use as thermal barrier coatings, further broadening their applicability across automotive, chemical processing, metallurgical, aerospace, and aviation industries [27].

Electrochemical impedance spectroscopy (EIS) and potentiodynamic polarization techniques are powerful tools for investigating the corrosion mechanisms of boride layers [15,28]. In HCl-containing acidic media, boriding has been shown to substantially reduce corrosion rates in steels such as AISI 304 and AISI 316L [29]. The corrosion behavior and surface characterization of alloys with compositions similar to Fe13Cr6Al have also been examined, underscoring the significance of microstructural changes within the surface layer [28]. FeCrAl alloys are also susceptible to brittle fracture during manufacturing and service [14], and efforts to strengthen their microstructure through surface engineering remain an active area of research [30]. Nevertheless, comprehensive studies on the electrochemical behavior of boride layers formed specifically on Fe20Cr5Al alloy in acidic environments remain scarce.

Despite extensive studies reporting that boriding enhances the corrosion resistance of conventional steels in acidic and chloride-containing media, the electrochemical behavior of boride layers formed on alumina-forming FeCrAl alloys, whose corrosion protection critically depends on the surface Al and Cr, remains unexplored. To the best of the authors’ knowledge, this is the first study to systematically investigate pack-boriding of Fe-20Cr-5Al ferritic alloy, with particular emphasis on the Al-repulsion mechanism and its consequences for passive film formation and corrosion resistance in acidic media. The present study addresses this gap with the following specific objectives: (i) to characterize the phase composition, nanocrystalline structure, and microstructure of the boride layer using XRD, Williamson-Hall analysis, and SEM/EDS; (ii) to quantify the mechanical enhancement in terms of surface hardness and wear rate; and (iii) to evaluate and mechanistically explain the effect of boriding on corrosion behavior in 5 wt.% H_2_SO_4_ through open-circuit potential (OCP), potentiodynamic polarization, and EIS measurements. In contrast to the protective behavior commonly reported for borided steels, this work demonstrates and mechanistically explains a boriding-induced loss of passivation specific to alumina-forming alloys.

## 2. Experimental Procedures

### 2.1. Substrate Material

The substrate material used in this study was an Fe-20Cr-5Al ferritic stainless-steel alloy. Specimens measuring 10 × 10 × 1 mm^3^ were prepared for surface treatment and subsequent characterization. The nominal chemical composition of the alloy is provided in Table 1.

### 2.2. Pack-Boriding Process

The boriding treatment was carried out in a stainless-steel (AISI 304) container measuring 30 × 30 × 50 mm^3^. Prior to processing, all specimen surfaces were ultrasonicated in ethanol to remove surface contaminants. Ekabor^®^-2 powder mixture (5 wt.% B_4_C + 5 wt.% KBF_4_ + 90 wt.% SiC; Bortec GmbH & Co. KG, Cologne, Germany) served as the boron source, and Ekrit powder (Bortec GmbH & Co. KG, Cologne, Germany) was used as the deoxidizer. The specimens were placed vertically in the boriding atmosphere, followed by the application of a 15-mm-thick deoxidizer layer on top of them. The container was then sealed to prevent atmospheric contamination. The boriding process was performed in a PID-controlled high-temperature furnace at 950 °C for 4 h, consistent with the optimum conditions reported in the literature for iron-based alloys [31]. After treatment, specimens were allowed to cool in the furnace for 8 h to minimize thermal shock and residual stress.

### 2.3. Structural and Microstructural Characterization

Surface morphology and cross-sectional microstructure were examined using an FEI Quanta 450 scanning electron microscope (FEI Company, Brno, Czech Republic). Elemental composition and spatial distribution were determined using an integrated Oxford X-MaxN 80 EDS system (Oxford Instruments, Abingdon, Oxfordshire, UK), including point analysis and elemental mapping across the boride layer cross-section.

The crystallographic phase composition of the boride layer was determined by XRD using Cu-Kα radiation (λ = 1.5406 Å) over a 2θ scanning range of 30–90° [16,32]. Phase identification was performed by comparing the diffraction patterns against standard reference cards. The average crystallite size (*D*) of the boride phases was calculated using the Scherrer equation (Equation (1)) [33], where β is the full width at half maximum (FWHM) of the diffraction peak in radians, θ is the Bragg angle, *K* is the dimensionless shape factor (0.94), and *λ* is the X-ray wavelength. Microstrain (ε) was independently estimated using Equation (2), where *β* and *θ* retain the same definitions. Peak positions and FWHM values were extracted using the profile-fitting function in PANalytical X’Pert HighScore Plus software (version 3.05, Malvern Panalytical, Almelo, The Netherlands). Only well-resolved, high-intensity Bragg reflections were selected; weak and overlapping peaks were excluded. The measured FWHM values were converted to radians prior to calculation. A Williamson-Hall (W-H) plot of *β* cos *θ* versus 4 sin *θ* was constructed for the five selected peaks, and linear regression was used to determine D from the y-intercept and ε from the slope. No instrumental broadening correction was applied; the reported crystallite size values should therefore be regarded as upper-bound estimates.

(1) $$D=\frac{K\lambda }{Bcos\;\theta \;}\;\;$$

(2) $$\varepsilon =\frac{\beta }{4tan\;\theta \;}\;\;$$

### 2.4. Microhardness and Wear Testing

Vickers microhardness measurements were performed using a HIGHWOOD HWMMT-X device (Highwood Co., Ltd., Osaka, Japan) equipped with a Vickers indenter tip, applying a 100-gf load with a 15-s dwell time. Measurements were taken from the surface toward the substrate interior, and average values were calculated for each indentation series. The hardness profiles of Fe20Cr5Al and Fe20Cr5Al-B specimens were compared to quantify the effect of the boriding treatment. Wear tests were performed using the TRIBOtechnic-TRIBOtester device (TRIBOtechnic, Clichy, France) at a load of 12 N, a total sliding distance of 250 m, and a sliding speed of 12 mm/s using Si_3_N_4_ balls as the counterpart material. The coefficient of friction (CoF) was recorded continuously throughout the test by the data acquisition system, and the friction profiles of both specimen groups were compared to assess the influence of surface modification on wear mechanisms. The cross-sectional areas of the wear tracks were measured using a Taylor Hobson 2D profilometer (Taylor Hobson Ltd., Leicester, UK). The tests were performed at 32–38% relative humidity and 23–28 °C. Wear volume (Equation (3)) and wear rate (Equation (4)) were calculated from measured wear-track data [34].

(3) V=A.l

(4) $$WR=\frac{V}{S}$$

where;

- *V*: Wear volume (mm^3^), *S*: Sliding distance (m),
- *A*: Area of the worn path (mm^2^), *l*: Wear Length (mm),
- *WR*: Wear Rate (mm^3^/m).

### 2.5. Electrochemical Testing

**Figure 1:**
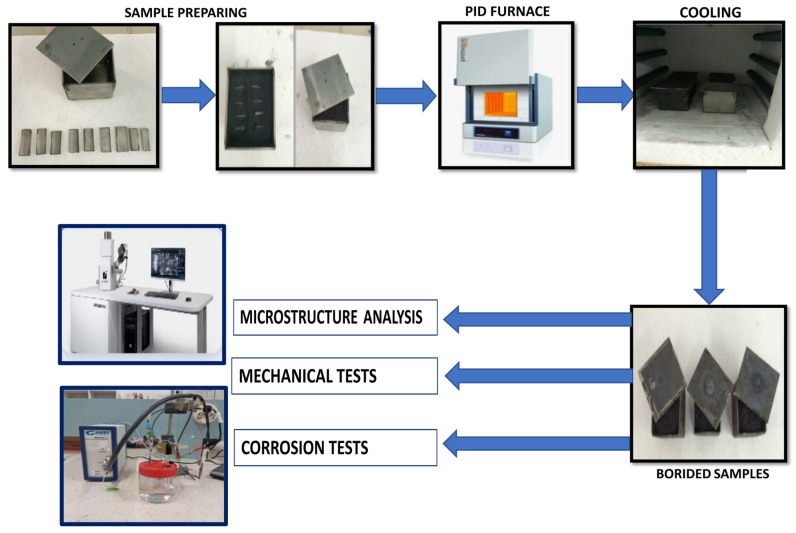
Schematic illustration of the pack-boriding setup and sequential characterization procedure for Fe20Cr5Al and Fe20Cr5Al-B specimens. Blue arrows indicate the direction of process flow.

Electrochemical tests were performed using a Gamry Reference 1010E potentiostat (Gamry Instruments, Warminster, PA, USA) at 26 ± 2 °C in a 5 wt.% H_2_SO_4_ solution using a standard three-electrode cell configuration. Sulfuric acid (5 wt.% H_2_SO_4_) was selected as the test electrolyte because it is representative of acidic condensate environments encountered in exhaust and flue gas systems and is widely used as a benchmark medium for evaluating the corrosion resistance of borided steels in the literature [25]. The working electrode consisted of the test specimen with an exposed surface area of 1.4 cm^2^; a platinum wire and an Ag/AgCl electrode were used as the counter and reference electrodes, respectively. Open-circuit potential (OCP) measurements were recorded over 3600 s to ensure steady-state conditions prior to electrochemical measurements [23]. Potentiodynamic polarization curves were recorded over a potential range of OCP ± 250 mV at a scan rate of 1 mV s^−1^. The corrosion potential (*E_corr_*) and corrosion current density (*I_corr_*) were determined by Tafel extrapolation [24,26]. Each stage of the test process, from sample preparation to application, is clarified in the schematic flow diagram shown in Figure 1. This visual approach enhances clarity of the experimental methodology and ensures test reproducibility. The corrosion rate (Equation (5)) was calculated using the following formula [35]:

(5) $$CR=k\frac{I_{corr\;}EW}{\rho }$$

where;

*k* = 3.27 × 10^−3^, constant, *I_corr_* = corrosion current density (µA.cm^−2^), *EW* = equivalent weight (g.eq^−1^), and *ρ* = experimental density (g.cm^−3^).

## 3. Results and Discussion

### 3.1. XRD Analysis

**Figure 2:**
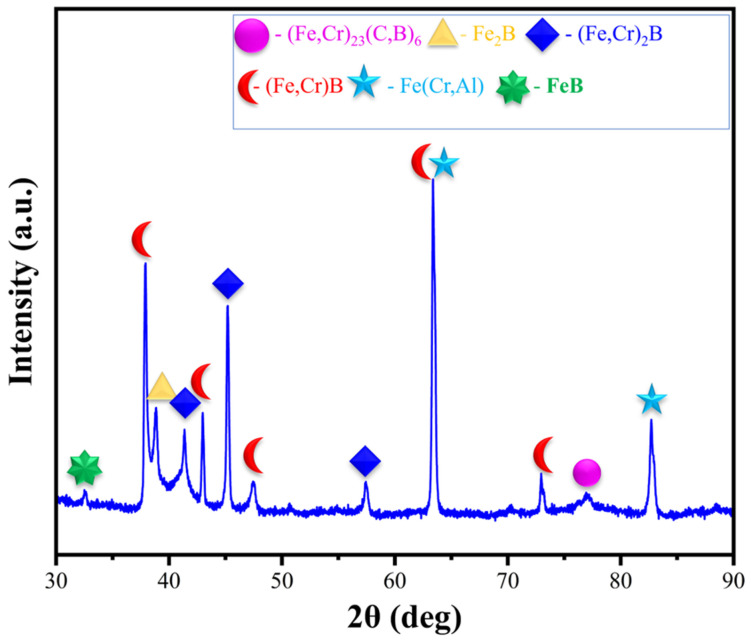
XRD pattern of Fe20Cr5Al-B with identified boride and carboboride phases.

The XRD diffraction pattern obtained from the surface of the pack-borided Fe20Cr5Al-B specimen is presented in Figure 2. The diffraction pattern reveals a multi-phase boride architecture rather than a homogeneous single-phase layer, reflecting the progressive decrease in boron concentration from the surface toward the substrate. The dominant phase identified at the surface is orthorhombic (Fe,Cr)B, with its most intense reflections observed at 2θ ≈ 37.87°, 41.32°, and 45.16°, corresponding to the (101), (111), and (200) crystallographic planes, respectively. A low-intensity reflection at 2θ ≈ 33° is tentatively attributed to FeB, though its weak signal places it near the noise threshold and should be interpreted with caution.

The formation of mixed (Fe,Cr)B and (Fe,Cr)_2_B borides in high-chromium borided steels is well established in the literature [36]. The near-identical atomic radii of Fe and Cr (~1.24 Å and ~1.25 Å, respectively) facilitate the substitution of Cr into the FeB/Fe_2_B crystal lattice without significant structural distortion, yielding (Fe,Cr)xB solid-solution phases. Consistent with previous reports [37], no independent CrB reflections were observed; instead, Cr is incorporated into the iron boride lattice substitutionally, with the slight angular shifts at 37.87° and 41.32° attributed to this lattice substitution effect.

The tetragonal (Fe,Cr)_2_B phase was identified by characteristic reflections at 2θ ≈ 40.5° and 43.8°, while Fe_2_B contributions are observed at 2θ ≈ 39.5°, with significant peak overlap between these structurally related phases at these positions. This two-phase gradient is consistent with boride growth thermodynamics: maximum boron activity at the outermost surface promotes (Fe,Cr)B nucleation, while the progressive decrease in boron activity toward the substrate drives a phase transition to the more thermodynamically stable (Fe,Cr)_2_B [38]. The comparatively lower peak intensities of (Fe,Cr)_2_B relative to the monoboride phase confirm that the surface layer within the X-ray penetration depth is predominantly composed of the (Fe,Cr)B zone [39].

A secondary reflection at 2θ ≈ 57.5° is attributed to the (Fe,Cr)_2_B phase, while the reflection at 2θ ≈ 75.5° is assigned to (Fe,Cr)_23_(C,B)_6_ carboboride phases in the diffusion zone, consistent with the carbon push-ahead mechanism during multi-component boriding. This phase, referred to in the literature as the Tau-phase, forms preferentially at the boride layer–substrate interface in chromium-rich systems due to Cr segregation [40]. Its presence provides evidence of the chemical gradient across the transition zone.

The most intense reflection at 2θ ≈ 63.5° is attributed to the overlapping contributions of the (Fe,Cr)B (002) plane and the α-Fe(Cr,Al) substrate reflection, while the peak at 2θ ≈ 83° is assigned to the α-Fe(Cr,Al) BCC substrate, confirming that X-rays penetrate through the boride layer into the underlying substrate at higher diffraction angles [41,42].

Rietveld refinement analysis performed using PANalytical X’Pert HighScore Plus software yielded quantitative phase fractions of 51.9 wt.% (Fe,Cr)B, 46.1 wt.% Fe_2_B, and 2.0 wt.% (Fe,Cr)_23_(C,B)_6_. The theoretical density of the boride layer, calculated from the refined weight fractions using the rule of mixtures (ρ(Fe,Cr)B = 7.48 g cm^−3^, ρFe_2_B = 7.33 g cm^−3^, ρ(Fe,Cr)_23_(C,B)_6_ = 6.97 g cm^−3^), was determined as 7.40 g cm^−3^ and applied in the corrosion rate calculations presented in Section 3.4.

To evaluate the nanoscale structural characteristics of the identified boride phases, Williamson-Hall (W-H) analysis was performed on the five most prominent diffraction peaks of the Fe20Cr5Al-B specimen. The average crystallite sizes (〈D〉) and microstrain (〈ε〉) were determined using Equation (1) [33] and Equation (2), respectively, and the results are summarized in Table 2. The analysis yielded an average crystallite size of 50.7 nm and a microstrain of 1.686 × 10^−3^, confirming the nanocrystalline nature of the boride phases formed during pack-boriding. The low microstrain value indicates minimal lattice distortion within the boride layer, suggesting that, despite the coating’s multiphase character, the individual boride crystallites exhibit a well-ordered internal structure. These findings are consistent with the sharp, well-defined diffraction peaks observed in Figure 2.

### 3.2. Microstructural Characterization

#### 3.2.1. Phase Distribution, Cross-Sectional Morphology and Elemental Analysis

**Figure 3:**
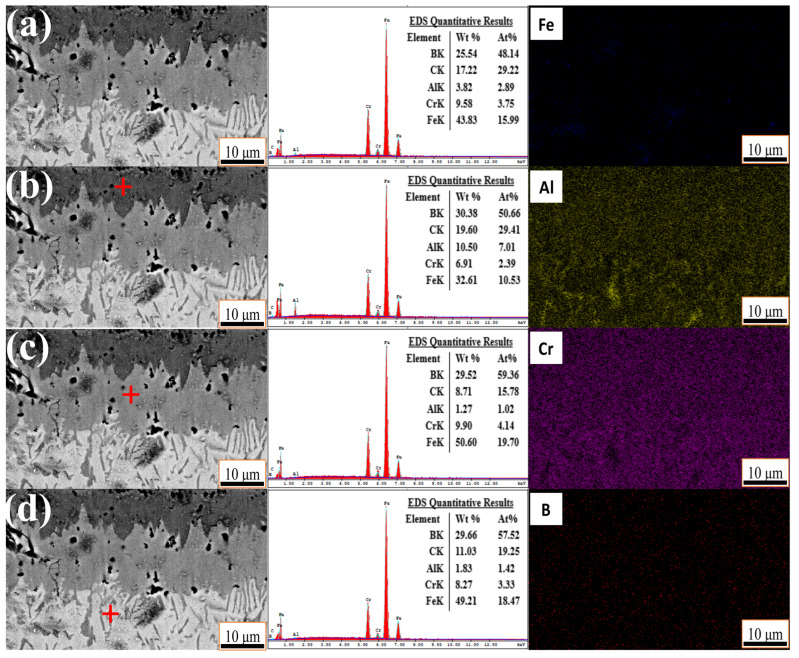
Cross-sectional SEM micrographs and EDS point analyses from selected regions (**a**–**d**) of the pack-borided Fe20Cr5Al-B specimen. Red cross (+) symbols indicate the exact locations of EDS point analysis measurements. EDS elemental maps (Fe, Al, Cr, B) shown in the right column were acquired from the general cross-sectional area presented in (**a**).

Figure 3 presents cross-sectional SEM micrographs and EDS point analyses from four distinct regions of the pack-borided Fe20Cr5Al-B specimen, alongside EDS elemental maps (Fe, Al, Cr, B) acquired from the general cross-sectional area shown in Figure 3a, providing comprehensive compositional evidence for the multi-phase boride layer architecture identified by XRD. The overall cross-section shown in Figure 3a reveals a complex, heterogeneous phase structure rather than a uniform single-layer coating, with distinct regions exhibiting different morphologies and contrast levels in backscattered electron imaging. The EDS spectrum collected from this region confirms the co-presence of B (25.54 wt.%), C (17.22 wt.%), Al (3.82 wt.%), Cr (9.58 wt.%), and Fe (43.83 wt.%), consistent with the overlapping boride and carboboride phases identified in the XRD pattern. The Fe elemental map shows reduced signal intensity within the boride layer relative to the substrate, reflecting Fe consumption during the formation of (Fe,Cr)B and (Fe,Cr)_2_B phases [36].

Figure 3b corresponds to the outermost region of the boride layer, appearing as a dark-grey contrast zone in the SEM micrograph. The EDS analysis of this region reveals elevated boron (30.38 wt.%) and carbon (19.60 wt.%) concentrations, along with a notably high aluminum content (10.50 wt.%). The enrichment of Al at the outermost surface is attributed to the Al-repulsion mechanism, whereby Al atoms are expelled from the advancing boride front and accumulate at the surface during boriding. This region is compositionally consistent with the (Fe,Cr)B phase, which XRD identifies as the dominant surface phase. The region analyzed in Figure 3c corresponds to the intermediate columnar layer beneath the outer monoboride zone, exhibiting a lighter grey contrast and well-developed columnar grain morphology. Compared with Figure 3b, the carbon content decreases markedly (by 8.71 wt.%), as does the aluminum concentration (by 1.27 wt.%), while the chromium content increases to 9.90 wt.%. This compositional profile is consistent with the (Fe,Cr)_2_B phase, in which Cr progressively substitutes Fe within the tetragonal Fe_2_B lattice as boron activity decreases with depth [36,37].

Figure 3d shows the boride-layer-substrate interface, characterized by acicular crystals penetrating the matrix. This transition zone exhibits residual carbon (11.03 wt.%) and aluminum (1.83 wt.%) concentrations, providing direct compositional evidence for both the carbon push-ahead mechanism and Al-repulsion operating concurrently during boriding [40]. The B elemental map confirms a pronounced columnar B-enriched zone with characteristic tooth-like morphology, consistent with the dominant boride phases identified by XRD.

**Figure 4:**
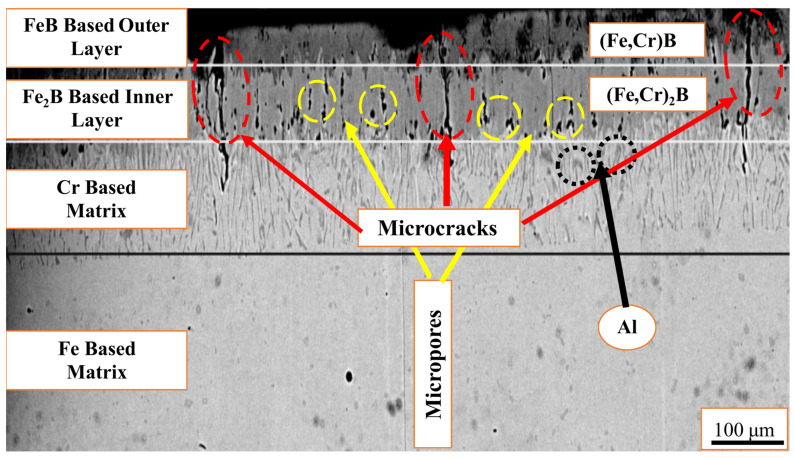
Cross-sectional SEM micrograph of Fe20Cr5Al-B showing the layered boride structure. Yellow dashed circles and yellow arrows: micropores; red dashed circles and red arrows: micro-cracks; black dashed circle and black arrow: Al-enriched transition zone.

The cross-sectional SEM micrograph of the Fe20Cr5Al-B specimen presented in Figure 4 reveals a layered boride architecture developing from the surface toward the substrate in the sequence: (Fe,Cr)B→(Fe,Cr)_2_B → acicular transition zone→Fe20Cr5Al matrix. The interface between the (Fe,Cr)B and (Fe,Cr)_2_B layers exhibits a sawtooth interlocking morphology, whereas the boundary between the (Fe,Cr)_2_B zone and the acicular transition region is comparatively planar.

The contrast variation across the cross-section reflects differences in mean atomic number (Z-contrast): bright regions correspond to Fe- and Cr-rich areas, while dark regions indicate zones enriched in low-atomic-number elements such as B and Al. The Al-enriched regions, highlighted in Figure 4, are spatially confined to the acicular transition zone and the substrate interface, providing further confirmation of the Al-repulsion mechanism identified in the EDS mapping analysis.

Notably, micropores are observed within the (Fe,Cr)_2_B inner layer, as indicated in Figure 4. These pores develop during boriding due to volumetric expansion associated with boride phase formation and the Kirkendall effect arising from differential diffusion rates of Fe and B. The presence of interconnected micropores within the boride layer creates preferential pathways for electrolyte penetration, contributing to the micro-galvanic cell formation and the deterioration in corrosion resistance discussed in the electrochemical analysis.

**Figure 5:**
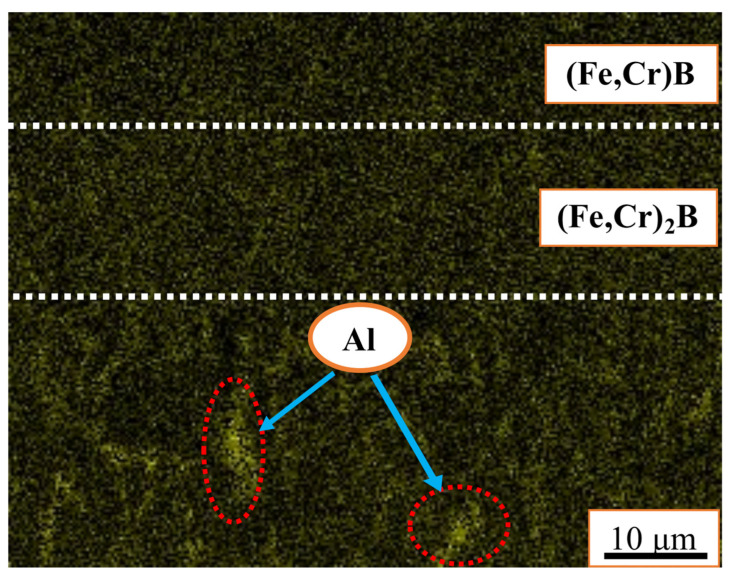
Al Kα elemental map of Fe20Cr5Al-B. White dashed lines indicate layer boundaries; red dashed circles and cyan arrows indicate Al-enriched regions.

Figure 5 presents the Al Kα elemental map of the Fe20Cr5Al-B cross-section. As shown in Figure 4, chromium concentrates preferentially beneath the (Fe,Cr)_2_B layer. The near identical atomic radius of Cr and Fe, combined with Cr’s high affinity for boron, allows chromium to be retained within the boride lattice rather than being expelled during layer growth, thereby supporting the formation of mixed (Fe,Cr)_2_B boride phases [36].

In contrast, aluminum exhibits the opposite behavior. The Al Kα map in Figure 5 reveals a gradual depletion of Al within the boride layer, with Al accumulating beneath the (Fe,Cr)_2_B zone and into the substrate. During interstitial diffusion of boron atoms, Al atoms are progressively displaced toward the deeper matrix regions, a phenomenon known as Al-repulsion [9,43,44]. This mechanistic contrast between Cr retention and Al expulsion within the boride layer is central to understanding the subsequent degradation of corrosion resistance in Fe20Cr5Al-B, as both Cr and Al are the primary passivation elements in the base alloy.

#### 3.2.2. EDS Line Scan and Elemental Depth Profile Analysis

**Figure 6:**
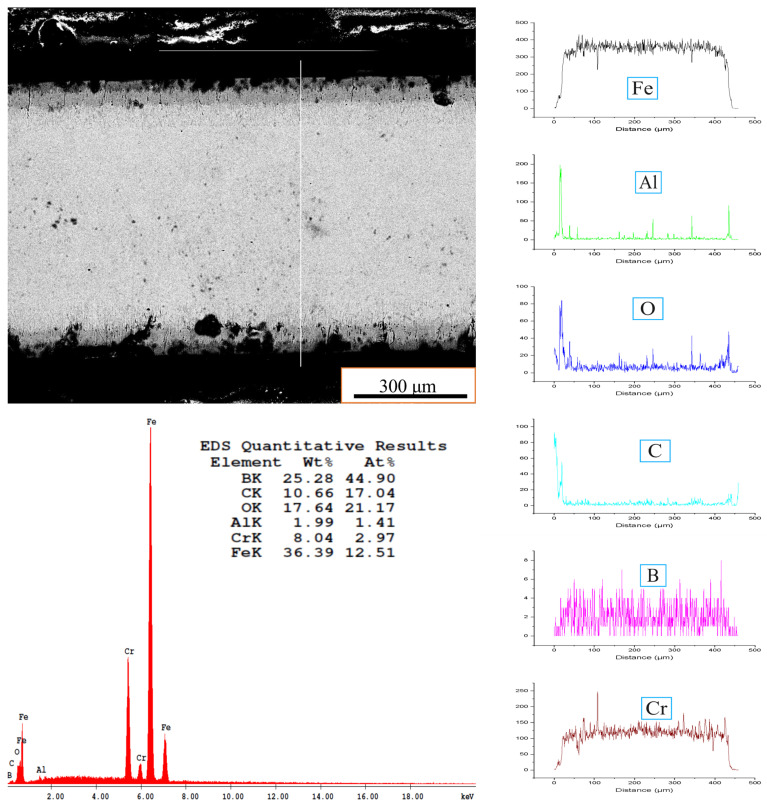
EDS line scan depth profile of the pack-borided Fe20Cr5Al-B specimen showing elemental distribution (Fe, Al, O, C, B, Cr) across the cross-section.

Figure 6 presents the EDS line scan analysis performed across the full cross-section of the pack-borided Fe20Cr5Al-B specimen, providing a compositional depth profile from the surface to the substrate. In the near-surface region (0–25 μm), elevated Al and O signals are observed simultaneously, suggesting a thin surface oxide layer, likely formed during cooling or sample preparation [8]. The relatively unstable B signal in this region is consistent with a barrier effect imposed by this oxide on boron diffusion. Beyond approximately 25 μm, Al and O signals decrease sharply while Fe, Cr, and B signals become dominant, marking the onset of the boride layer. The inverse relationship between Fe and B intensities at this depth confirms atomic-scale substitution of Fe by B during boride phase formation.

A characteristic Cr enrichment peak is observed at approximately 105–125 μm, coinciding with a local B maximum. This co-enrichment reflects the preferential clustering of (Fe,Cr)_2_B phases in this region, driven by Cr’s high affinity for boron [36,37]. Between 125 and 315 μm, the B signal exhibits oscillatory behavior consistent with the sawtooth boride morphology observed in SEM: signal maxima correspond to boride needles, while minima correspond to interstitial matrix regions. This interlocking morphology provides mechanical anchoring of the boride layer to the substrate [45].

Beyond 315 μm, the B signal diminishes, and Fe and Cr profiles stabilize toward bulk alloy composition, marking the diffusion zone boundary. Residual B traces detected up to approximately 425 μm confirm that boron diffusion extends well beyond the visible boride layer. To further interpret the elemental depth profiles, the diffusion kinetics of boron in the Fe20Cr5Al alloy during pack-boriding at 950 °C for 4 h were estimated using the Arrhenius relation and Fick’s second law [46]. The boron diffusion coefficient at 950 °C was calculated as D = 1.15 × 10^−11^ m^2^/s, using a pre-exponential factor D_0_ = 3.4 × 10^−3^ m^2^/s and activation energy Q = 199.16 kJ/mol for boron diffusion in iron-based systems [ref]. The theoretical maximum diffusion depth was estimated as x = 2√(Dt) ≈ 814 μm. The experimentally observed boride layer thickness of 80–85 μm is significantly lower than this value, attributed to the consumption of boron during boride phase formation, the retarding effect of Cr on boron diffusivity through preferential (Fe,Cr)B and (Fe,Cr)_2_B phase formation, and the partitioning of boron among multiple coexisting phases.

#### 3.2.3. Surface Morphology and Defect Analysis

**Figure 7:**
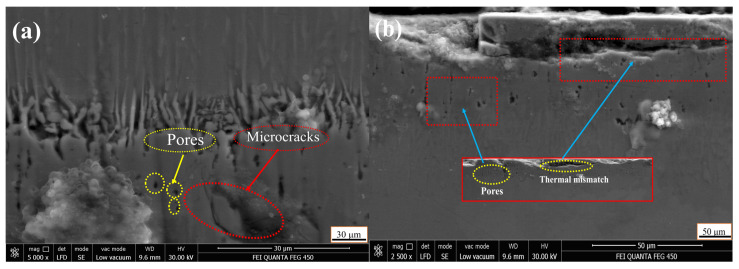
SE (**a**) and BSE (**b**) SEM images of the top surface and cross-section of Fe20Cr5Al-B. In (**a**): yellow dashed circles indicate pores; red dashed circles indicate micro-cracks. In (**b**): the yellow dashed circle indicates pores; the red rectangles highlight regions of interest, with the inset showing thermal mismatch-induced cracking; the cyan arrows indicate pores and thermal mismatch regions.

Figure 7 presents SE (a) and BSE (b) SEM images of the top surface and cross-section borided Fe20Cr5Al-B specimen at different magnifications, revealing both surface topography and cross-sectional phase contrast. The SE images show that boron diffusion did not proceed uniformly across the surface. Localized protrusions developed at preferential nucleation sites where boride growth was more aggressive, resulting in a heterogeneous surface topography. Fine micro-cracks are visible on the boride surface at higher magnification, attributed to the thermal expansion coefficient (CTE) mismatch between the hard, brittle boride layer and the ductile Fe20Cr5Al substrate. During cooling, the inability of the boride layer to accommodate the strain imposed by the substrate leads to the accumulation of tensile stress and the formation of microcracks [47]. Scattered micro-voids are also observed, interpreted as Kirkendall porosity arising from the differential diffusion rates of Fe and B atoms during boriding [36]. Image analysis of BSE-SEM cross-sectional micrographs using ImageJ/Fiji software (version 2.14.0/1.54f, National Institutes of Health, Bethesda, MD, USA) revealed a total porosity of 7.6–14.8% and a crack density of up to 33 × 10^−3^/μm within the (Fe,Cr)_2_B layer, with round Kirkendall-type pores (AR ≤ 2.0) constituting the dominant defect morphology (>53%) and the longest detected micro-crack reaching 21.8 μm corresponding to near-through-thickness penetration of the (Fe,Cr)_2_B zone.As discussed in the electrochemical analysis, these micro-cracks and pores serve as preferential pathways for electrolyte penetration, significantly compromising corrosion resistance.

The BSE images in Figure 7b provide compositional contrast across the cross-section. Dark regions correspond to areas enriched in low-atomic-number elements (B and Al), while bright regions represent Fe- and Cr-rich zones. The progressive displacement of Al toward the substrate interior, visible as an Al-depleted dark halo beneath the boride layer, is consistent with the Al-repulsion mechanism identified in the EDS analyses. This Al depletion not only modifies the surface composition but also undermines the alloy’s oxidation resistance by eliminating its ability to form Al_2_O_3_ at the surface [9,43,44,48].

### 3.3. Microhardness and Wear Measurements

**Figure 8:**
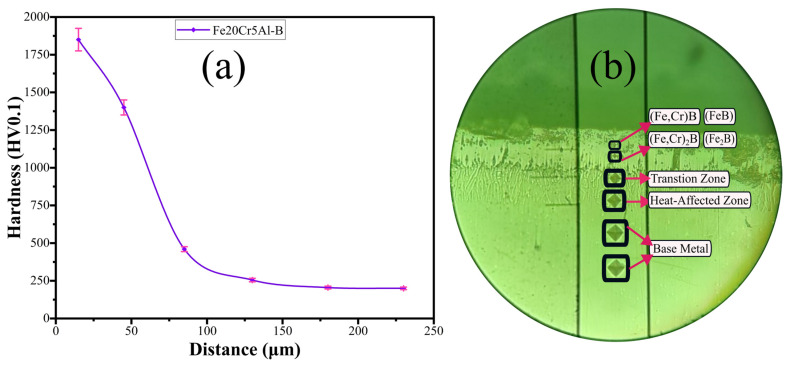
Vickers microhardness depth profile (**a**) and optical micrograph of indentation marks (**b**) for Fe20Cr5Al-B.

Figure 8 presents the Vickers microhardness depth profile (a) and the corresponding optical micrograph of indentation marks (b) for the Fe20Cr5Al-B specimen, measured under a 100-gf load from the surface toward the substrate interior. The highest hardness value of 1854 HV was recorded at the outermost surface (indent 1), where the (Fe,Cr)B phase is dominant. This value reflects not only the intrinsic hardness of the FeB-type monoboride but also the solid-solution hardening contribution of Cr substituting Fe within the boride lattice, forming (Fe,Cr)B and (Fe,Cr)_2_B phases harder than their binary Fe-B counterparts [36,37]. The second indent (1402 HV) corresponds to the inner (Fe,Cr)_2_B layer, where the reduced boron content yields a somewhat lower but still exceptionally high hardness. The sharp drop at indent 3 (464 HV) marks the transition zone, where boride crystal growth terminates, and boron exists primarily in solid solution within the diffusion zone. Beyond this point, hardness stabilizes between 200 and 250 HV (indents 4–6), consistent with the characteristic hardness of the Fe20Cr5Al base alloy [40,49]. The effective case depth, defined as the depth at which hardness drops below 450 HV, is approximately 80–85 μm, consistent with the columnar boride morphology observed in SEM. Overall, pack-boriding increased the surface hardness of Fe20Cr5Al by approximately 9-fold relative to the untreated alloy.

**Figure 9:**
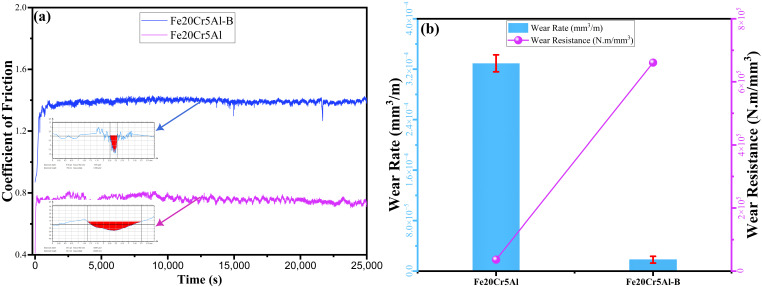
(**a**) CoF profiles for Fe20Cr5Al and Fe20Cr5Al-B, with cross-sectional wear-track profilometry insets. Red areas in the profilometry insets represent the measured cross-sectional wear-track area used to calculate wear volume. and (**b**) wear rate and wear resistance comparison for Fe20Cr5Al and Fe20Cr5Al-B under dry sliding conditions.

Figure 9 presents the coefficient of friction (CoF) profiles (a) and wear rate/wear resistance comparison (b) for Fe20Cr5Al and Fe20Cr5Al-B under dry sliding conditions against a Si_3_N_4_ counterpart. The CoF of Fe20Cr5Al stabilized at approximately 0.76 throughout the test, reflecting the dominance of adhesive wear on the unborided alloy surface. In contrast, Fe20Cr5Al-B exhibited a lower and stable CoF of approximately 0.65, representing a 14.5% reduction relative to the unborided alloy. The reduction in CoF is attributed to the formation of a tribofilm at the boride surface during sliding, which acts as a solid lubricant and reduces adhesive interactions with the Si_3_N_4_ counterpart. The high hardness of the (Fe,Cr)B and (Fe,Cr)_2_B phases resists plastic deformation at the contact interface, shifting the dominant wear mechanism from adhesive to abrasive and thereby yielding higher friction values [50,51]. Despite the increase in CoF, the borided specimen demonstrated markedly superior wear performance: the wear rate decreased from 3.29 × 10^−4^ to 1.82 × 10^−5^ mm^3^/m an approximately 18-fold reduction, while wear resistance increased correspondingly from 36,439 to 660,793 N·m/mm^3^ [36,52].

The stable friction profile of Fe20Cr5Al-B, in contrast to the fluctuating CoF of the unborided alloy, confirms that the boride layer acts as an effective tribological barrier, suppressing material loss despite the higher contact stress. The duplex (Fe,Cr)B + (Fe,Cr)_2_B layer architecture plays a critical role here: the hard outer monoboride phase provides wear resistance, while the underlying (Fe,Cr)_2_B layer, being comparatively ductile, prevents catastrophic delamination by accommodating subsurface stress [36,51,52].

To contextualize these results within the broader boriding literature, Table 3 presents a comparative summary of the mechanical, tribological, and corrosion properties of the borided Fe20Cr5Al-B alloy alongside selected borided tool steels and stainless steels. The borided Fe20Cr5Al-B specimen exhibits a wear rate comparable to that of borided AISI H13 and AISI D2 tool steels, demonstrating the competitive tribological performance of the developed boride layer. Notably, in contrast to borided stainless steels such as AISI 304 and 316L, which exhibit improved corrosion resistance after boriding, the Fe20Cr5Al-B alloy shows deteriorated corrosion behavior, underscoring the unique role of the Al-repulsion mechanism in alumina-forming alloy systems.

### 3.4. Electrochemical Corrosion Analysis

#### 3.4.1. Open Circuit Potential (OCP)

**Figure 10:**
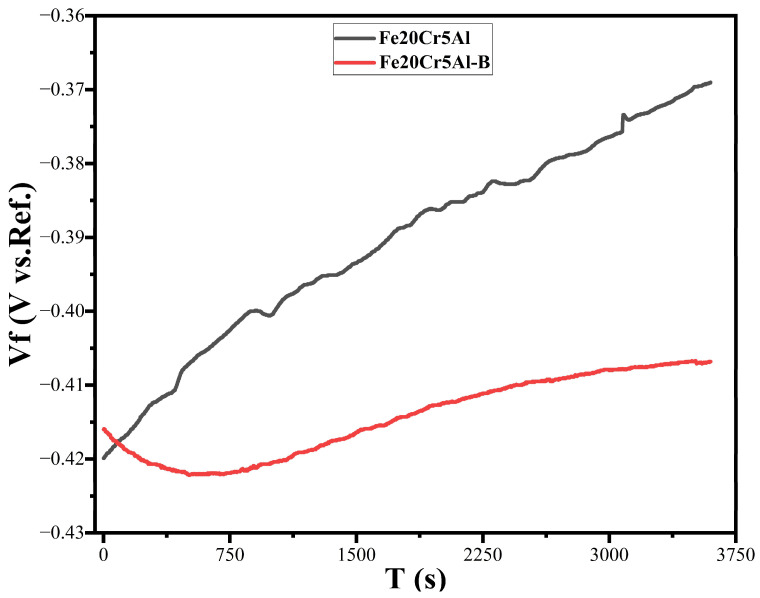
OCP vs. time curves for Fe20Cr5Al and Fe20Cr5Al-B in 5 wt.% H_2_SO_4_.

Figure 10 presents the evolution of the open-circuit potential (OCP) over 3600 s for Fe20Cr5Al and Fe20Cr5Al-B in 5 wt.% H_2_SO_4_ solution. The OCP of Fe20Cr5Al shifted steadily in the positive direction from approximately −0.42 V to −0.370 V (vs. Ag/AgCl), indicating progressive surface stabilization through the formation of a thin passive film, primarily Cr_2_O_3_ and Al_2_O_3,_ that resists complete dissolution in the acidic medium [36,54]. The continuous positive drift reflects the gradual growth and consolidation of this protective oxide layer over time.

In contrast, Fe20Cr5Al-B exhibited a slight initial cathodic shift from −0.416 V to a minimum of approximately −0.423 V, followed by a gradual recovery to −0.413 V at steady state. This behavior indicates that the boride surface, while initially relatively stable upon immersion, becomes mildly activated as the acidic electrolyte penetrates the boride layer through microcracks and pores. The absence of a clear positive OCP drift in Fe20Cr5Al-B, unlike the pronounced passivation trend observed in Fe20Cr5Al, confirms that the Al-repulsion mechanism has depleted the surface of the Al and Cr necessary for passive film formation, leaving the boride layer susceptible to active dissolution in H_2_SO_4_.

#### 3.4.2. Potentiodynamic Polarization Analysis

**Figure 11:**
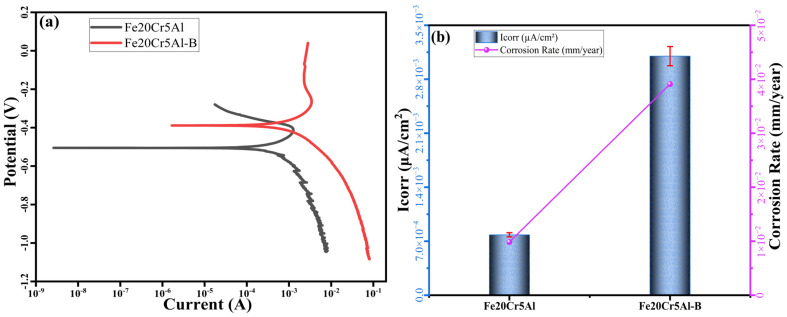
(**a**) Polarization curves, (**b**) *I_corr_* and corrosion rate comparison for Fe20Cr5Al and Fe20Cr5Al-B.

Figure 11 presents the potentiodynamic polarization curves (a) and the corresponding *I_corr_* and corrosion rate comparison (b) for Fe20Cr5Al and Fe20Cr5Al-B in 5 wt.% H_2_SO_4_. The corrosion potential (*E_corr_*) of Fe20Cr5Al was measured at −0.459 V and shifted to −0.295 V after boriding, a positive (noble) shift of approximately 164 mV. While this thermodynamic shift might suggest reduced surface reactivity, the corrosion kinetics tell a different story. The corrosion current density (*I_corr_*) increased from 7.83 × 10^−4^ to 3.10 × 10^−3^ mA cm^−2^, corresponding to a ~4-fold increase in corrosion rate from 9.67 × 10^−3^ to 3.83 × 10^−2^ mm/year (Table 4). This apparent contradiction between a nobler *E_corr_* and higher *I_corr_* is a hallmark of galvanic or structural discontinuity-driven corrosion rather than true passivation [54,55,56].

The superior corrosion resistance of Fe20Cr5Al is attributed to the spontaneous formation of a highly protective, thermodynamically stable passive film composed of Al_2_O_3_ and Cr_2_O_3_ on the alloy surface [57,58]. Boriding disrupts this passive film formation through two concurrent mechanisms: the Al-repulsion effect depletes the surface of Al and Cr, the primary passivation elements, while micro-cracks and Kirkendall pores within the boride layer create preferential pathways for electrolyte penetration, accelerating active dissolution. As a result, the borided surface, despite its ceramic character, fails to maintain a coherent passive barrier in H_2_SO_4_, and instead undergoes accelerated corrosion driven by micro-galvanic interactions at phase boundaries [59,60].

Corrosion rates were calculated according to ASTM G102, using an equivalent weight of 27.92 g/eq and a density of 7.40 g/cm^3^ for the Fe20Cr5Al alloy composition. Corrosion rates were calculated in accordance with ASTM G102, using an equivalent weight of 27.92 g/eq. The density of Fe20Cr5Al was taken as 7.25 g/cm^3^, while the theoretical density of the boride layer (Fe20Cr5Al-B) was determined as 7.40 g/cm^3^ from Rietveld refinement (Section 3.1). All electrochemical parameters are summarized in Table 4.

**Figure 12:**
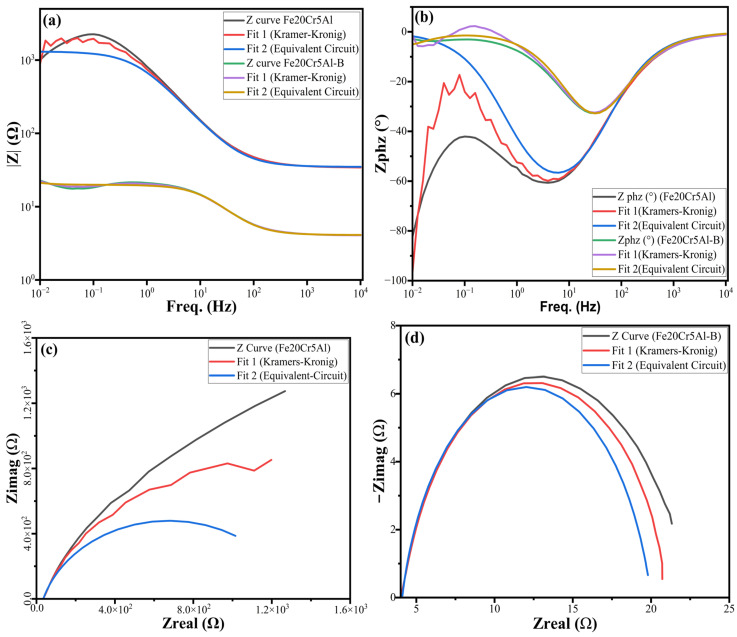
Bode impedance modulus (**a**), Bode phase angle (**b**), and Nyquist diagrams (**c**,**d**) of Fe20Cr5Al and Fe20Cr5Al-B with Kramers-Kronig validation and equivalent circuit fits.

Figure 12 presents the EIS results for Fe20Cr5Al and Fe20Cr5Al-B in 5 wt.% H_2_SO_4_, including Bode impedance modulus (a), Bode phase angle (b), and Nyquist diagrams for Fe20Cr5Al (c) and Fe20Cr5Al-B (d), with experimental data, Kramers-Kronig validation, and equivalent circuit fits. Fe20Cr5Al was modeled using an R_s_(Q_dl_R_ct_) circuit, while Fe20Cr5Al-B required a two-time-constant R_1_(Q_2_[R_4_(Q_5_R_7_)]) circuit, consistent with the more complex interfacial response of the defective boride layer.

The Bode impedance modulus plot (Figure 12a) reveals that Fe20Cr5Al exhibits |Z| values of 1000–2000 Ω in the low-frequency region, confirming the strong barrier character of the naturally formed Al_2_O_3_/Cr_2_O_3_ passive film. In contrast, Fe20Cr5Al-B shows a dramatic reduction in |Z| to 15–20 Ω across the measured frequency range, indicating that the boride layer offers negligible resistance to ionic transport in the aggressive H_2_SO_4_ environment. The Bode phase angle plot (Figure 12b) shows that Fe20Cr5Al maintains a stable phase angle between −60° and −80° across a wide frequency range, indicating a capacitive, corrosion-resistant passive layer. The phase angle of Fe20Cr5Al-B reaches a maximum of approximately −32° before approaching 0° at low frequencies, reflecting a transition from capacitive to resistive behavior attributed to electrolyte penetration through micro-crack and porosity networks within the boride layer.

The Nyquist diagram of Fe20Cr5Al (Figure 12c) shows a large capacitive arc with a polarization resistance (R_p_) of approximately 1282 Ω, confirming charge-transfer-controlled corrosion kinetics governed by the intact passive film. The Nyquist diagram of Fe20Cr5Al-B (Figure 12d) reveals a dramatically reduced arc diameter, with a total polarization resistance of only R_p_ = R_4_ + R_7_ = 15.87 + 4.08 = 19.95 Ω. The low CPE exponent (*n* = 0.844) confirms that the boride surface deviates significantly from ideal capacitive behavior, consistent with a rough, porous, and heterogeneous microstructure. These findings are fully consistent with the potentiodynamic polarization results and confirm that the Al-repulsion mechanism, combined with micro-cracks and Kirkendall porosity networks, is responsible for the severe deterioration in corrosion resistance following pack-boriding.

#### 3.4.3. Effect of Corrosion on the Borided Surface

**Figure 13:**
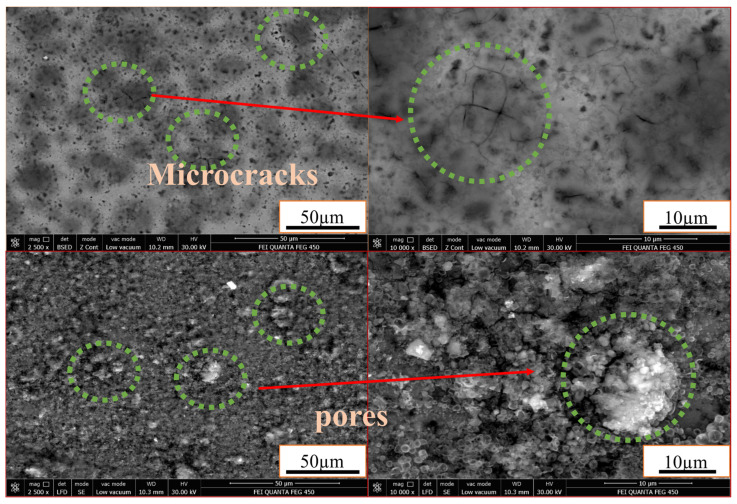
SEM images of Fe20Cr5Al-B after corrosion testing showing micro-cracks (**top**, BSED) and pores (**bottom**, SE) at 2500× and 10,000× magnification. Green dashed circles indicate regions of interest; red arrows show the specific region magnified in the right panels.

Figure 13 presents post-corrosion SEM images of the Fe20Cr5Al-B surface at two magnifications (2500× and 10,000×) in both BSED and SE modes, revealing the structural defects responsible for the observed deterioration in corrosion resistance.

The low-magnification BSED images show a network of micro-cracks traversing the boride surface. At higher magnification, these cracks reveal an interconnected character that provides direct electrolyte access to the underlying substrate, effectively bypassing the boride layer and acting as preferential corrosion pathways in H_2_SO_4_ [36,53,61]. The SE images in the lower panels confirm the presence of scattered pores distributed across the surface, exhibiting a rounded morphology consistent with Kirkendall porosity formed during boriding. At higher magnification, these pores appear as sites of localized dissolution, where electrolyte accumulation accelerates localized corrosion.

The deterioration in corrosion resistance is not solely structural. As established by the EDS analyses, Al and Cr atoms are expelled from the boride lattice during layer growth, creating an Al-depleted zone at the surface. Under normal conditions, micro-crack tips would be rapidly repassivated by the formation of an Al_2_O_3_ and Cr_2_O_3_ film; however, the depletion of both elements from the borided surface eliminates this self-healing capability. Consequently, fresh metal exposed at crack tips comes into direct contact with the aggressive electrolyte, promoting undermining corrosion and eventual spalling of the boride layer [8,48,62,63,64,65].

## 4. Conclusions

This study examined the structural, mechanical, and electrochemical characteristics of the boride layer formed on an Fe-20Cr-5Al ferritic alloy via pack boriding at 950 °C for 4 h. The following conclusions can be drawn:

XRD analysis confirmed the formation of a hierarchical, multi-phase boride layer consisting of orthorhombic (Fe,Cr)B as the dominant surface phase, tetragonal (Fe,Cr)_2_B in the inner zone, and (Fe,Cr)_23_(C,B)_6_ carboboride phases in the diffusion zone, attributed to the carbon push-ahead mechanism. Rietveld refinement yielded a phase fraction of 51.9 wt.% (Fe,Cr)B, 46.1 wt.% Fe_2_B, and 2.0 wt.% (Fe,Cr)_23_(C,B)_6_, with a theoretical boride layer density of 7.40 g/cm^3^. Williamson–Hall analysis yielded an average crystallite size of 50.7 nm and a microstrain of 1.686 × 10^−3^, confirming the nanocrystalline character of the boride phases.

SEM/EDS analyses revealed a sawtooth boride morphology approximately 80–85 μm in thickness, with elemental mapping providing direct evidence for both the Al-repulsion mechanism manifested as near-zero Al signal within the boride zone and the carbon push-ahead effect, which drives (Fe,Cr)_23_(C,B)_6_ formation at the diffusion front. Quantitative image analysis revealed a porosity of 7.6–14.8% and a crack density of up to 33 × 10^−3^ μm^−1^ within the (Fe,Cr)_2_B layer.

Pack-boriding increased the surface hardness of Fe20Cr5Al approximately 9-fold, reaching 1854 HV (18.18 GPa) at the outermost (Fe,Cr)B zone. The hardness declined systematically with depth through the (Fe,Cr)_2_B layer (1402 HV) and transition zone (464 HV), stabilizing at 200–250 HV in the substrate, consistent with the base alloy hardness.

Tribological testing demonstrated an ~18-fold reduction in wear rate (from 3.29 × 10^−4^ to 1.82 × 10^−5^ mm^3^/m) and a corresponding ~18-fold increase in wear resistance, confirming the effectiveness of the boride layer as a tribological barrier. The coefficient of friction decreased from 0.76 to 0.65 (a 14.5% reduction), attributed to tribofilm formation on the boride surface during sliding.

Electrochemical analyses in 5 wt.% H_2_SO_4_ revealed a paradoxical deterioration in corrosion resistance following boriding. Despite a noble shift in *E_corr_* from −0.459 to −0.295 V, the corrosion current density increased from 7.83 × 10^−4^ to 3.10 × 10^−3^ mA cm^−2^, and the corrosion rate increased ~4-fold, from 9.67 × 10^−3^ to 3.83 × 10^−2^ mm/year. EIS analysis confirmed a dramatic reduction in total polarization resistance from 1282 to 19.95 Ω. This deterioration is attributed to the synergistic effect of Al-repulsion, which eliminates the Al_2_O_3_/Cr_2_O_3-_based passive film formation capability and microcrack/porosity networks that facilitate electrolyte penetration and micro-galvanic cell formation.

These findings demonstrate that while pack-boriding is highly effective for tribological enhancement of FeCrAl alloys, achieving concurrent corrosion protection requires optimizing the integrity of the boride layer by minimizing microcracks and porosity. Future work will focus on evaluating the borided layer in additional corrosive environments, including chloride-containing and neutral-pH media, and on developing post-boriding surface treatments and modified boriding parameters to simultaneously enhance wear and corrosion performance.

## Figures and Tables

**Table 1:** Chemical composition of the Fe20Cr5Al alloy (wt.%).

	C	Si	Mn	Cr	Al	Fe
**Min**	-	-	-	20.5	-	-
**Max**	0.08	0.7	0.5	23.5	-	-
**Nominal**	-	-	-	-	4.8	Bal.

**Table 2:** Crystallite size (〈D〉) and microstrain (〈ε〉) of the boride phases in pack-borided Fe20Cr5Al-B alloy determined by Williamson-Hall analysis.

Peak No	2θ (°)	FWHM (°)	〈D〉 (nm)	〈ε〉 (×10^−3^)
7	37.886	0.188	46.6	2.384
10	42.958	0.145	61.4	1.607
14	45.164	0.167	54.0	1.750
28	63.375	0.202	48.2	1.428
57	82.690	0.253	43.6	1.259

**Table 3:** Comparison of pack-borided Fe20Cr5Al-B with selected borided steels and heat-resistant alloys: process conditions, mechanical, tribological, and corrosion properties.

Material	T/t	Thickness (μm)	Hardness (HV)	Wear Rate (mm^3^/m)	CoF	Corrosion	Ref
**Fe20Cr5Al-B**	950 °C/4 h	80–85	1854	1.82 × 10^−5^	0.65	H_2_SO_4_	This Work
**AISI H13**	1000 °C/8 h	N/R	1803	2.53 × 10^−5^	0.15–0.25	N/R	[53]
**AISI D2**	1000 °C/8 h	N/R	2322	2.02 × 10^−5^	0.15–0.25	N/R	[53]
**X165CrV12**	950 °C/9 h	~150	1800–2000	-	-	N/R	[36]

**Table 4:** Electrochemical parameters of Fe20Cr5Al and Fe20Cr5Al-B obtained by Tafel extrapolation.

Materials Code	*E_corr_* (mV)	*I_corr_* (µA/cm^2^)	Corrosion Rate (mm/Year)
Fe20Cr5Al	−0.459	0.783	9.67 × 10^−3^
Fe20Cr5Al-B	−0.295	3.100	3.83 × 10^−2^

## Data Availability

The original contributions presented in this study are included in the article. The raw data supporting the conclusions of this article are available on request from the corresponding author.
